# Three Empty Bottles and a Wide QRS: Rapid Alkalinization Rescue in Propafenone Overdose

**DOI:** 10.19102/icrm.2026.17065

**Published:** 2026-06-15

**Authors:** Afnan Chaudhry, Ibrahim Inanc, Saad Khan, George Prousi, Shilla Zachariah

**Affiliations:** 1 Phoenixville Hospital Tower Health, Phoenixville, PA, USA

**Keywords:** Alkalinization, class IC anti-arrhythmic, overdose, propafenone, toxicity

## Abstract

Propafenone is a class IC anti-arrhythmic agent with potent fast sodium channel blockade whose overdose may result in profound intraventricular conduction delay and malignant ventricular arrhythmias. Management strategies are largely derived from isolated case reports, and consensus guidelines are lacking. We describe a case of a young man with a history of supraventricular tachycardia who presented following intentional polysubstance ingestion, including at least 600 mg of propafenone. On presentation, he was hypoxemic, acidotic, and minimally responsive. Electrocardiography revealed sustained wide complex tachycardia with marked QRS widening. Initial laboratory testing demonstrated normal electrolyte levels with a modestly elevated troponin, along with severe anion-gap metabolic acidosis, elevated lactate levels, acute kidney injury, and toxic acetaminophen concentrations. Intravenous sodium bicarbonate was initiated to achieve mild alkalemia, along with aggressive electrolyte correction and supportive intensive care. Within 1 h of bicarbonate therapy, the ventricular tachycardia resolved with rapid narrowing of the QRS complex and restoration of sinus rhythm. Serial electrocardiograms over the ensuing 24 h showed continued normalization of conduction. This case highlights the electrophysiologic manifestations of propafenone toxicity and underscores the value of QRS duration as a dynamic marker of toxicity severity and treatment response. Early alkalinization with intravenous sodium bicarbonate may rapidly correct conduction disturbances and stabilize malignant arrhythmias in severe class IC anti-arrhythmic overdose.

## Introduction

Propafenone is a class IC anti-arrhythmic agent with potent fast sodium channel-blocking activity and additional β-adrenergic and calcium channel antagonist effects, commonly used for rhythm control in atrial fibrillation and other supraventricular tachyarrhythmias.^1,2^ Although propafenone toxicity is rare, overdose can produce profound conduction abnormalities and life-threatening arrhythmias. Various treatment strategies have been described in isolated case reports, but no standardized management guidelines exist yet.^3–8^

We describe a case of acute propafenone toxicity presenting with ventricular tachycardia and significant metabolic derangements following intentional polysubstance ingestion. This case illustrates the electrophysiologic manifestations of propafenone toxicity and highlights the effectiveness of early alkalinization therapy in reversing critical conduction disturbances.

## Case presentation

A male patient in his 20s with a history of dysautonomia, paroxysmal supraventricular tachycardia, connective tissue disease, chronic pain, and depression was found unresponsive next to empty bottles of propafenone, hydrocodone–acetaminophen, and lorazepam, along with a suicide note at 07:00 h. He had last been seen well the prior evening at 23:00 h. Emergency medical services reported an oxygen saturation of 62% and minimal responsiveness despite naloxone therapy, requiring emergent intubation upon arrival to the emergency department.

Institutional review board approval was not required for this study, as it represents a single-patient case report with no prospective intervention, research-related procedures, or identifiable patient information.

**Figure 1:**
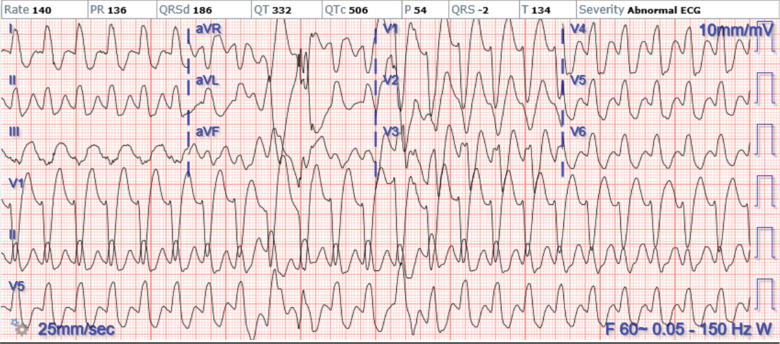
Initial 12-lead electrocardiogram (ECG) demonstrating severe propafenone toxicity. The ECG shows a wide-complex tachycardia at approximately 140 bpm with marked QRS prolongation (186 ms). There is pronounced intraventricular conduction delay with a sine-wave-like pattern, consistent with advanced sodium channel blockade. Subtle atrioventricular conduction is difficult to appreciate due to marked QRS widening. These findings represent classic electrophysiologic manifestations of class IC anti-arrhythmic overdose.

The initial electrocardiogram (ECG) revealed a sustained wide complex tachycardia at 140 bpm with pronounced QRS widening **(Figure 1)**. Laboratory evaluation demonstrated normal potassium levels, modestly elevated troponin, severe anion-gap metabolic acidosis, lactate elevation, acute kidney injury, toxic acetaminophen levels, and a urine toxicology screen positive for opiates **(Table 1)**. His family reported that the patient may have ingested at least 600 mg of propafenone, 29 hydrocodone–acetaminophen tablets (totaling an estimated 9.75 g of acetaminophen), and an unknown quantity of lorazepam. Given his normal potassium level and only modest troponin elevation, electrolyte abnormalities and acute coronary syndrome were considered less likely, and the arrhythmia was attributed to propafenone toxicity.

**Table 1:** Pertinent Laboratory Findings on Presentation

Laboratory Test	Value	Reference Range
High-sensitivity troponin I	149 ng/L	<53 ng/L
Potassium	4.5 mmol/L	3.5–5.1 mmol/L
Bicarbonate	13 mEq/L	20–31 mEq/L
Lactate	6.8 mmol/L	0.6–1.4 mmol/L
Acetaminophen level	73.5 μg/mL	<20 μg/mL
Creatinine	1.28 mg/dL	0.73–1.18 mg/dL
Urine toxicology	Positive for opiates	Negative

In consultation with Poison Control, immediate intravenous sodium bicarbonate infusion was initiated, targeting mild alkalemia (pH, 7.45–7.50). To further stabilize myocardial conduction, intravenous magnesium and comprehensive electrolyte repletion were provided for ventricular arrhythmia suppression. Given the significant acetaminophen ingestion, N-acetylcysteine therapy was also started. A low threshold was maintained for synchronized cardioversion should ventricular tachycardia re-emerge, and vasopressor therapy was anticipated if hemodynamic instability or hypotension occurred.

**Figure 2:**
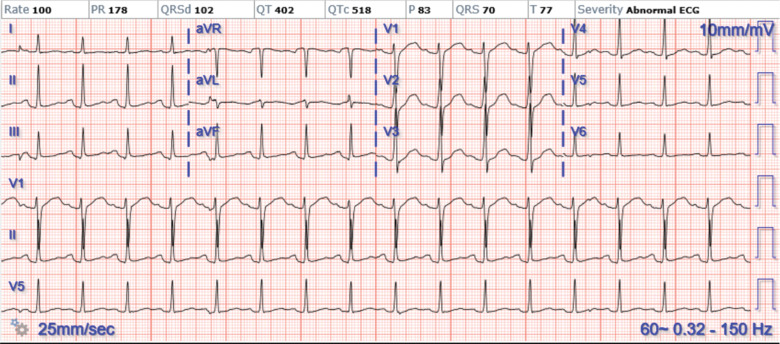
Follow-up 12-lead electrocardiogram (ECG) 1 h after initiation of intravenous sodium bicarbonate therapy. This ECG shows substantial improvement after bicarbonate therapy, with QRS narrowing from 186 to 102 ms and resolution of the prior sine-wave morphology. Sinus rhythm at ~100 bpm is restored with improved atrioventricular conduction.

Within 1 h of initiating intravenous sodium bicarbonate, the wide complex tachycardia resolved, converting to sinus tachycardia with progressive QRS narrowing **(Figure 2)**. Over the subsequent 24 h, serial ECGs and continuous telemetry demonstrated continued normalization of conduction with a narrow QRS. Transthoracic echocardiography revealed a hyperdynamic left ventricular ejection fraction (>75%) without structural abnormalities, findings consistent with reversible drug-induced myocardial and conduction toxicity. By the following day, the bicarbonate infusion was discontinued, and the patient was safely extubated and monitored on telemetry for an additional 24 h, during which he remained in stable sinus rhythm. This was accompanied by normalization of acetaminophen levels and preserved hepatic function. A comprehensive psychiatric evaluation was subsequently completed for intentional overdose and ongoing suicidality.

## Discussion

Propafenone toxicity represents a rare but clinically significant form of class IC anti-arrhythmic overdose, with the potential to produce profound conduction disturbances, hemodynamic instability, and malignant ventricular arrhythmias. This patient’s presentation of wide complex tachycardia, marked QRS prolongation, and significant metabolic derangements illustrates the characteristic electrophysiologic and systemic effects of potent sodium channel blockade. The rapid narrowing of the QRS and stabilization of rhythm following intravenous sodium bicarbonate highlight the role of early alkalinization in reversing propafenone-induced conduction toxicity. Collectively, these findings underscore the importance of prompt recognition of the characteristic ECG abnormalities of propafenone toxicity, which facilitates rapid toxicology involvement, vigilant monitoring, and timely initiation of sodium channel blockade reversal therapy.

The dose of propafenone required to produce toxicity is highly variable and cannot be reliably predicted by the reported ingestion amount, as extensive first-pass metabolism and inhibition of cytochrome P450 pathways can markedly amplify plasma concentrations, permitting severe and potentially life-threatening toxicity even at relatively modest doses.^9^ Propafenone also demonstrates a rate-dependent sodium channel-blocking effect, such that, during tachycardia, even therapeutic concentrations may manifest electrophysiologic toxicity with QRS widening and conduction failure, underscoring the importance of exercise or stress testing in selected patients to unmask clinically significant conduction abnormalities not apparent at rest.^10^ In this case, the patient’s complex metabolic derangements and sustained tachycardia likely contributed to severe toxicity despite an estimated ingestion of approximately 600 mg, although the true ingested dose may have been higher. Propafenone toxicity produces a characteristic pattern of conduction slowing through potent class IC sodium channel blockade, resulting in impaired phase 0 depolarization and delayed intraventricular conduction. At elevated serum concentrations, additional β-adrenergic and calcium channel-blocking properties may further exacerbate bradycardia, hypotension, and myocardial depression.^2,3^ As a result, propafenone overdose is associated with a broad spectrum of electrocardiographic abnormalities, including sinus bradycardia or arrest, atrial fibrillation, P–R prolongation, bundle branch block, and marked QRS and QT interval widening, with Brugada-pattern changes and ventricular tachycardia also described.^4–7^ In severe toxicity, progressive conduction delay may culminate in asystole or electromechanical dissociation, highlighting the lethal potential of advanced sodium channel blockade.^11–13^ Among these findings, QRS prolongation is the most dependable marker of toxicity severity and has repeatedly been shown to correlate with a higher risk of malignant ventricular arrhythmias.^4–7^

Within this electrophysiologic landscape, our patient exemplified the tachyarrhythmic end of the spectrum, presenting with sustained wide complex tachycardia at 140 bpm and significant QRS prolongation. Yet, prior literature illustrates how unpredictable the clinical phenotype can be. Haddad et al. described a far milder presentation characterized by P–R prolongation, left bundle branch block, and QRS widening without ventricular tachycardia, all of which resolved with hydration and discontinuation of propafenone.^4^ Gil et al. reported profound conduction slowing with extreme QRS widening, junctional rhythms, and a transient Brugada pattern but no sustained ventricular tachycardia.^5^ Other cases reveal an entirely different bradyarrhythmic profile, including the severe junctional bradycardia responsive to atropine and dopamine described by Alsaad et al. and the massive overdoses documented by Avci et al. and Ardiç et al., where chaotic ventricular rhythms or marked sinus bradycardia with extreme QRS widening required aggressive bicarbonate therapy, inotropic support, mechanical ventilation, and temporary pacing to achieve stabilization.^3,6,7^ Cardiac arrest has likewise been reported in fulminant toxicity.^8–10^ Despite these differing presentations, all reports share a unifying trajectory in which clinical recovery consistently parallels progressive narrowing of the QRS complex, underscoring the central value of monitoring QRS dynamics as a real-time marker of therapeutic response.

Although numerous cases of propafenone toxicity have been described, there remains no unified or evidence-based treatment strategy, and management is guided largely by physiologic principles. In our patient, intravenous sodium bicarbonate produced rapid QRS narrowing and rhythm stabilization, mirroring the effectiveness of alkalization in prior reports.^6,7^ The therapeutic benefit of sodium bicarbonate in propafenone and other class IC anti-arrhythmic toxicities is multifactorial. An increased serum sodium concentration competitively overcomes sodium channel blockade, while systemic alkalinization reduces drug binding to fast sodium channels and facilitates dissociation of the weakly acidic drug from myocardial tissue. Additionally, buffering of acidosis may protect end-organ function and further improve cardiac conduction. Together, these mechanisms support sodium bicarbonate as a cornerstone therapy in severe propafenone toxicity.^8^

However, not all presentations may respond promptly to bicarbonate alone. The use of intravenous lipid emulsion has also been described, suggesting a potential rescue therapy when conventional measures fail. However, not all presentations may respond promptly to bicarbonate alone. Refractory cases described in the literature have required escalation to temporary pacing, inotropic support, mechanical ventilation, and even extracorporeal membrane oxygenation. Intravenous lipid emulsion has also been used as a rescue modality.^11–13^ Collectively, these experiences underscore the absence of a standardized approach and reinforce the need for further research.

Importantly, this case adds several clinically meaningful insights to the existing literature. First, it provides a clear, high-resolution temporal correlation between intravenous sodium bicarbonate administration and rapid electrophysiological reversal, with dramatic QRS narrowing and termination of sustained wide complex tachycardia within 1 h, documented by serial ECGs. Second, it highlights QRS duration as a dynamic bedside marker of both toxicity severity and treatment response, rather than a static diagnostic finding—an aspect often noted but rarely demonstrated so clearly in published cases.^3–7^ Third, unlike many reports focusing on refractory or multimodal rescue therapies, this case demonstrates successful stabilization with early, targeted alkalinization alone.^11–13^

## Conclusion

This case demonstrates the range of potential arrhythmias and conduction disturbances associated with propafenone toxicity, including wide complex tachycardia and QRS widening. Although clinical presentations vary widely across reported cases, a consistent pattern emerges in which clinical deterioration and recovery follow the QRS complex, making QRS dynamics a valuable real-time marker of therapeutic response. In our patient, early recognition of the characteristic ECG abnormalities prompted timely administration of intravenous sodium bicarbonate, leading to rapid improvement in conduction and stabilization of rhythm. While bicarbonate therapy has been effective in our case and in several others, there is currently no standardized management strategy for propafenone overdose. Further research is needed to establish evidence-based guidelines and clarify the optimal approach to managing this rare but potentially life-threatening toxicity.
